# Supplementary material for: Nasal povidone-iodine implementation for preventing surgical site infections: Perspectives of surgical nurses
Source: PLoS One. 2020 Nov 19;15(11):e0242217. doi: 10.1371/journal.pone.0242217 (PMC7676708; doi:10.1371/journal.pone.0242217)
Supplement: S1 File — (PDF) [file pone.0242217.s001.pdf]

INTERVIEW GUIDE FOR NURSES TO EVALUATE PRE-SURGICAL NASAL POVIDONE-IODINE IMPLEMENTATION

Qualitative interview introduction

Length: 20-30 minutes

1. *Verbal consent:* The purpose of this interview is to understand your experiences and perspective regarding the pre-surgical nasal povidone-iodine (PI) solution application procedure that was recently initiated at the facility. This interview will last 20 to 30 minutes and will be audio recorded. This recording will be transcribed with all identifiers removed. The recording and transcript will be stored on a secure, password-protected server. Participation in this interview is completely voluntary. At any time during the interview you can decide you no longer wish to participate. A decision not to participate will not affect your employment in this facility in any way. Would you like to participate in this interview?
2. *Background information*:
   - (i) Name
   - (ii) Profession
   - (iii) How long have you been working in your current position?
3. Please tell me about the process of starting to use PI prior to surgery was like in your clinic
   a. Definite probes:
      i. How were you told about the change?
      ii. When were you told about the change?
      iii. What were you told about the change?
      iv. Was there any information about the change that you didn't get ahead of time that you wish you would have?
      v. What did you think about the written protocol? Anything missing? Anything not make sense? (if yes, how did they address gaps in understanding?)

vi. What did you think about how the change was handled or communicated to you?

4. When you first learned that this change was going to happen, what did you think about it?

5. Could you please walk me through the process of applying PI pre-surgically?

   a. Possible probes:

      i. What do you do first? After that?

      ii. Approximately how many minutes does it take you to finish applying PI for both nostril?

      iii. After the PI application, approximately how many minutes does it take before a patient is sent for surgery?

      iv. Did you obtain verbal consent from the patient/ explained the PI procedure to the patient?

   b. Definite probes:

      i. What types of patients do you normally administer the PI to?

      ii. What challenges did you encounter with this process?

      iii. Were there times that you were unable to complete this process as you've just described it to me? (interruptions, patient objections, due to known allergy)

      iv. Did you notice any gaps in how the PI was administered at your clinic?

      v. From your perspective, how did the PI application impact patients?

         1. What questions or comments did they have?

         2. What do you think they thought of it?

         3. Were there any adverse effects that you noticed?

         4. If yes, what did you do recuse the patient's condition? And what was the outcome?

6. What training or instruction did you receive on how to apply the povidone-iodine? What do you think about this training?

    Probe:

    i. Did you receive enough information or direction that helped you to perform your duty effectively?
    ii. If not, what do you think can be done to improve the training?
    iii. How many days was the training?

7. Now that the PI process has been going on for a few months, what do you think about it?
    a. Probe:
        i. In what ways could this process be improved?

8. Is there anything else I haven't asked you about that you think it's important for me to know about this topic?

9. How would you rate the success of the PI implementation process, using a scale of 1 (Poor) to 10 (excellent)?

THANK YOU FOR YOUR TIME.
